# Increased suPAR Plasma Levels May Indicate Postoperative Sepsis Following Open Thoracoabdominal Aortic Repair

**DOI:** 10.3390/jcm14248843

**Published:** 2025-12-14

**Authors:** Dragos Socol, Cathryn Bassett, Bernhard Hruschka, Jelle Frankort, Moustafa Elfeky, Katja Heller, Florian Kahles, Berkan Kurt, Christian Uhl, Panagiotis Doukas, Alexander Gombert

**Affiliations:** 1 Faculty of Medicine, RWTH Aachen University, Campus Aachen, 52062 Aachen, Germany; 2 Department of Vascular Surgery, European Vascular Center Aachen-Maastricht, RWTH University Hospital Aachen, 52074 Aachen, Germany; 3 Department of Internal Medicine I—Cardiology, University Hospital Aachen, 52074 Aachen, Germany

**Keywords:** open thoracoabdominal aortic aneurysm, suPAR, sepsis, acute kidney injury

## Abstract

**Background/Objectives**: Postoperative organ complications following open thoracoabdominal aortic aneurysm (TAAA) repair pose significant challenges during the early postoperative period, where prompt detection is crucial for improving patient outcomes. Sepsis is often a central factor in these complications. This study investigates the perioperative dynamics of soluble urokinase plasminogen activator receptor (suPAR) plasma levels in TAAA patients undergoing elective surgical repair and evaluates its diagnostic potential for early detection of postoperative sepsis. **Methods**: In this retrospective, single-center study, 28 patients (mean age 52.6 ± 13.4 years; 67.9% male) underwent elective open TAAA repair between 2022 and 2024. Blood samples were collected at five perioperative time points, and suPAR levels were measured using ELISA. The primary endpoint was the onset of postoperative sepsis, with secondary endpoints including other organ complications. The predictive performance of suPAR levels was evaluated using Receiver Operator Characteristics (ROC) analysis. **Results**: Postoperative sepsis developed in 7 of 28 patients (25%), with the diagnostic criteria met at a mean of 9.7 ± 6.9 days. Baseline suPAR levels did not differ between groups; however, from 12 h after surgery, the sepsis group exhibited significantly higher serum concentrations (14.43 ng/mL vs. 7.23 ng/mL; *p* = 0.004), a difference that persisted throughout the first 24 h. At 24 h, suPAR had the highest predictive accuracy for sepsis, with an AUC of 0.90, 90% sensitivity, and 86% specificity at a 9 ng/mL cut-off (*p* < 0.001). **Conclusions**: Elevated suPAR levels in the early postoperative period are strongly associated with the later onset of sepsis. Early monitoring may enable timely intervention, potentially improving outcomes in this high-risk patient population.

## 1. Introduction

Thoracoabdominal aortic aneurysms (TAAAs) pose a significant clinical challenge, which if left untreated might lead to increased mortality and morbidity. While endovascular techniques have revolutionized TAAA management, leading to a substantial decrease in open repair procedures being performed [1], open surgery remains indispensable for complex anatomies, infections, failed endovascular interventions, and, often, patients with connective tissue disorders [2,3,4].

Open TAAA repair is a major surgical procedure, with potential postoperative dysfunction of multiple organ systems [3,5,6]. Postoperative complications, including acute respiratory distress syndrome (ARDS), acute kidney injury (AKI), multiple-organ dysfunction syndrome (MODS) or sepsis as a common underlying mechanism, contribute to a 30-day mortality rate of 12% following open TAAA repair. The above-mentioned complications affect up to 37% of patients [3]. Early identification of these complications, particularly sepsis, is crucial for improving patient outcomes.

Currently, a vast array of biomarkers has been proposed to aid in the detection of sepsis [7]. Previous research has explored the correlation between biomarker levels and specific complications like ARDS (Biologically Active Adrenomedullin) [8], AKI (Neutrophil Gelatinase-associated Lipocalin) [9] and visceral ischemia syndrome (Intestinal Fatty Acid Binding Protein) [10]. Among emerging biomarkers, the soluble form of the urokinase plasminogen activator receptor (suPAR) holds promise, as it has been found to specifically identify patients at risk for postoperative sepsis, independent of specific organ failures [11]. SuPAR’s role in immunomodulation, particularly in the activation and recruitment of neutrophils and macrophages, makes it a potentially valuable tool in the early detection of sepsis [12].

Originally, suPAR was shown to be a robust prognostic marker in the treatment of various carcinoma (breast, colorectal, ovarian and prostate cancer) [13] and autoimmune diseases [14]. Previous studies investigated the possible use of suPAR measurements in aiding the prediction of postoperative complications after noncardiovascular surgery [15]. Nonetheless, studies tackling the perioperative dynamics of the suPAR concentration following major cardiovascular interventions are limited and do not focus on a possible correlation between the suPAR concentration following open TAAA repair and the development of sepsis.

Established biomarkers (CRP and procalcitonin) used in the diagnosis of sepsis have been shown to be elevated during the acute sepsis phase, reaching their maximal concentration at 48 h after the initial trigger [16,17]. In comparison to established biomarkers, elevated suPAR levels might allow for early stratification of patients prone to develop sepsis in the early postoperative phase due to being an integral part of the inflammation cascade, as well as a marker for endothelial integrity [18].

The aim of this study is to evaluate the accuracy and predictive power of elevated serum suPAR levels as an early biomarker for sepsis in the immediate postoperative period following open TAAA surgery.

## 2. Materials and Methods

### 2.1. Study Design

**Figure 1:**
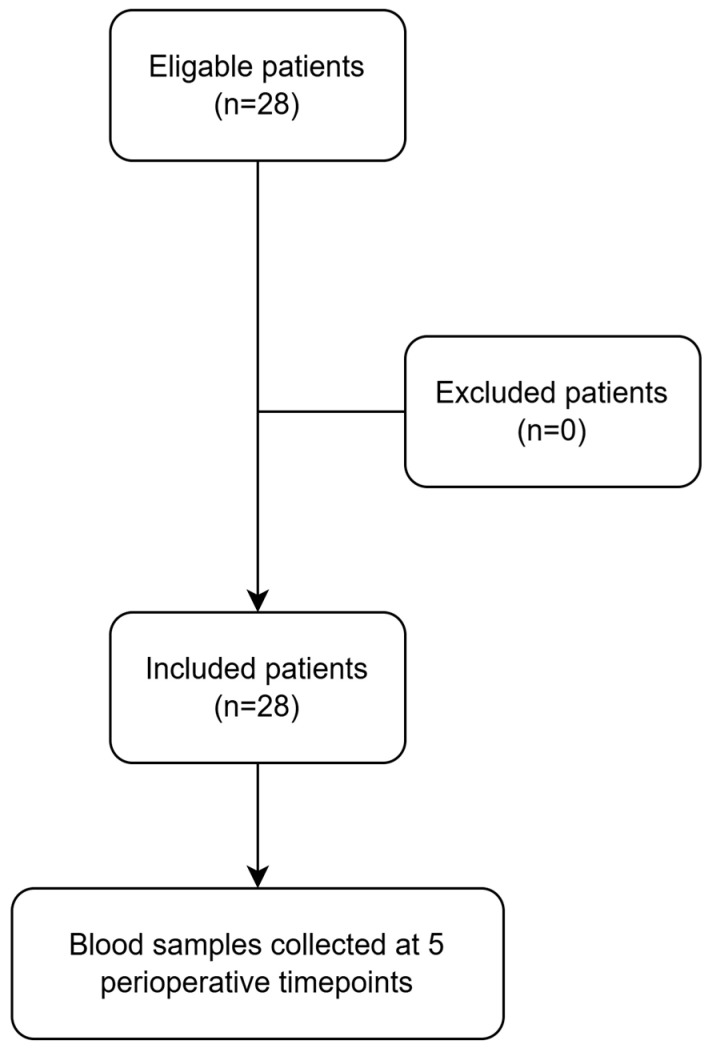
Patient Flowchart.

This retrospective study was designed in accordance with the STROBE criteria and the Declaration of Helsinki, and it was approved by the Ethics Committee of the University Hospital Aachen (EK010/19, approval date: 1 July 2024). The details on patient recruitment and material acquisition were preregistered as part of a wider research project at clinicaltrials.gov (NCT04087161). We present a single-center, observational trial, with a cohort consisting of 28 consecutively enrolled patients that underwent an elective TAAA operation, according to the Crawford criteria, at the University Hospital Aachen, between May 2020 and June 2024, treated by the same surgeon respecting standardized operative procedures. The patients’ medical records were analyzed through the IntelliSpace Critical Care and Anesthesia (ICCA) and MEDICAL management software (Cloud Software Group Switzerland GmbH, Schaffhausen, Switzerland). The patients that have been included in our study received the intervention consecutively, without being selected from a larger cohort, thus minimizing selection bias. We included all 28 initially selected participants, after applying exclusion criteria such as age < 18 years, already present bacteriemia, ASA > 3, emergent cases or intraoperative mortality. The study selection process is presented in Figure 1. Written informed consent was obtained from all patients prior to enrolment and surgery.

### 2.2. Material Acquisition

Arterial blood samples were collected from the patients at five distinct timepoints (preoperative, directly postoperative, and 12, 24, and 48 h after surgery). After collection, the blood samples were centrifuged at 3000 rpm and 4° Celsius for 10 min. The samples were then aliquoted and stored at −80° Celsius, as prior studies confirmed that freezing at −80° Celsius and subsequent thawing did not affect the long-term integrity of suPAR, validating the ability to measure cryogenically frozen samples [19]. The samples were analyzed twice separately, using an enzyme-linked immunosorbent assay kit for detecting serum suPAR concentrations with a sensitivity of 0.094 ng/mL and a detection range of 0.156 ng/mL–10 ng/mL, following the specified conditions of use (ABIN6971303, antibodies-online GmbH, Aachen, Germany). In order to not exceed the detection limit of the ELISA kits, a dilution of 1/10 of the samples was used for the measurements and no measurements exceeded the detection range.

### 2.3. Surgery

The surgical steps of the TAAA reconstruction surgery and the difficulties in postoperative management have previously been described in the specialized literature [20]. The aim of the intervention is to replace the affected aortic segment with a synthetic graft while also maintaining the perfusion of the connected vessels, such as the superior mesenteric artery, the celiac trunk, as well as the renal arteries. The procedure entails exposure of the thoracoabdominal aortic wall through a thoracolaparotomy, followed by placing a femoral-femoral cannula through the femoral vessels for cardiopulmonary bypass. After clamping of the proximal aorta and during the reconstruction of the visceral segment, no-flow time in the periphery is bridged with the use of extracorporeal circulation, with a flow of at least 500 mL/min. CUSTODIOL^®^ solution (Dr. Franz Köhler Chemie GmbH, Bensheim, Germany) was used in kidney perfusion in order to minimize the cellular activity and protect the organ integrity during the operation. The goal of the procedure is the connection of a graft to the still healthy normal aortic tissue above the aneurismatic segment [21].

Additionally, the following protective measures aimed at preserving the function of the spinal cord have been implemented in each intervention: cerebrospinal fluid drainage, motor evoked potential monitoring and moderate hypothermia [22,23].

### 2.4. Endpoints

The primary endpoint was the correlation between the concentration differences in serum suPAR pre- and post-surgery and the clinical diagnosis of sepsis. Sepsis was defined in accordance with “The Third International Consensus Definitions for Sepsis and Septic Shock”, as an increase of at least 2 points on the Sequential Organ Failure Assessment (SOFA) score in the span of a day and the clinical suspicion of an active infection [24].

Further secondary endpoints included postoperative organ complications. AKI was defined using the KDIGO criteria, as a postoperative increase of over 26.5 mol/L in serum creatinine or 1.5 times of the baseline value at 48 h post-surgery, with the KDIGO stage 3 (minimum 3 times creatinine level increase) necessitating the use of kidney replacement therapy being classified as a relevant complication [25]. ARDS was diagnosed in accordance with the Berlin criteria, as PaO_2_:FIO_2_ < 300 mmHg or SpO_2_:FIO_2_ < 315 on HFNO with flow of >30 L/min or NIV/CPAP with at least 5 cm H_2_O end-expiratory pressure and severe ARDS was classified as a relevant complication [26].

### 2.5. Statistics

Categorical variables are expressed as absolute frequencies and percentages. Continuous variables with a normal distribution are reported as mean ± standard deviation, whereas those with heavy skewness are presented as median [interquartile range]. Statistical significance was defined as *p* ≤ 0.05 with a 95% confidence interval. Categorical comparisons were performed using Fisher’s exact test, Pearson’s correlation was used for normally distributed continuous variables, and the Mann–Whitney test was applied for nonparametric continuous data. The accuracy of suPAR blood levels at each of the five timepoints for identifying at-risk individuals was evaluated using Receiver Operating Characteristic (ROC) analysis, with the Area Under the Curve (AUC) calculated after log-transforming the suPAR concentrations to achieve a normalized distribution. The optimal cut-off point, which provided the best balance between sensitivity and specificity, was determined using the Youden Index. Survival analysis was performed using a Cox proportional hazards regression model to identify predictors for sepsis during hospital stay. Time-to-event was measured in days. Predictor variables entered into the Cox model were suPAR levels at 12 h after surgery, duration of surgery and the difference in CRP levels during the first postoperative day. Predictors were selected based on statistically significant associations in previous assessments. Results are presented as hazard ratios (HR) with corresponding 95% confidence intervals (95% CI) and *p*-values. For statistical testing, suPAR concentrations were log-transformed to approximate a normal distribution; however, all reported values in tables and figures represent raw (non-transformed) concentrations for clinical interpretability. The low event rate could impact the robustness of a multivariable regression analysis, as such a model could have been statistically underpowered. We focused our analysis on univariable comparisons and the temporal association of suPAR dynamics with sepsis onset. ROC analyses were conducted using the log-transformed suPAR values, and the optimal cut-off for each measurement timepoint was determined using the Youden Index. All statistical analyses were performed using SPSS software version 29 (SPSS Inc., Chicago, IL, USA), and all graphical representations were produced with GraphPad Prism version 10.3.0 for Windows (GraphPad Software, San Diego, CA, USA).

## 3. Results

### 3.1. Preoperative Characteristics

Out of the 28 included patients with a mean age of 52.6 ± 13.4 years, 67.9% were men and had a mean maximal aneurysm diameter of 6.46 ± 1.45 cm before the procedure. A total of 7 (25%) patients developed sepsis postoperatively. A detailed analysis of the demographic characteristics and perioperative data of the patients is shown in Table 1. Several preexistent conditions were present in a higher proportion in the group of sepsis patients, e.g., chronic kidney disease (71% vs. 42%, *p* = 0.385), diabetes mellitus (29% vs. 5%, *p* = 0.145) and previous aortic operations in the medical history (71% vs. 2%, *p* = 0.67), although none of them reached a statistically significant difference between the sepsis and non-sepsis group.

### 3.2. Postoperative Parameters

The sepsis group experienced a significantly longer median hospital stay compared to non-sepsis patients (55 days vs. 25 days, *p* = 0.028). The need for catecholamines was also higher in sepsis patients (20 vs. 2 days *p* = 0.042). The group of sepsis patients showed higher percentages of postoperative complications (ARDS, pneumonia, AKI or post operative delirium), while across the total population no statistical significance was reached with the probability of developing sepsis in the postoperative phase (Table 2).

SuPAR concentration levels in the postoperative phase showed an increase in the total study population until 12 h postoperatively in comparison to the baseline levels (8 ng/mL versus 2.54 ng/mL). Until the last recording at 48 h postoperatively, the blood levels of suPAR began to gradually decrease (6.83 (4.97–12.02) ng/mL).

**Figure 2:**
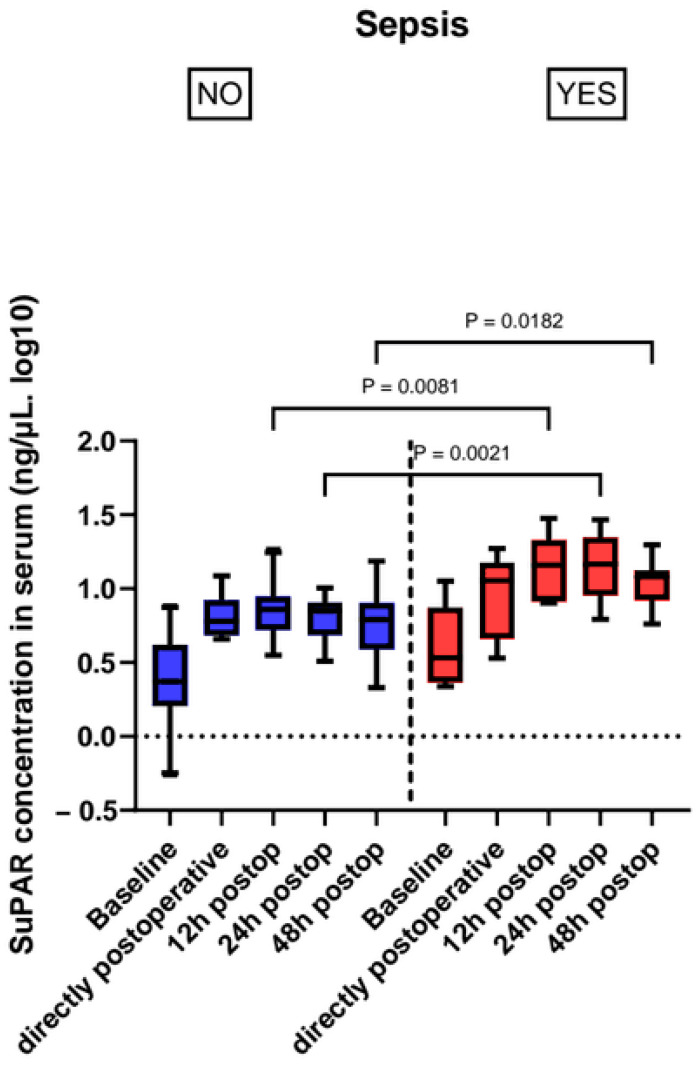
Perioperative suPAR levels in patients with sepsis and without sepsis.

We found higher suPAR levels in patients that became septic during their ICU stay, showing statistical significance from the 12 h timepoint (14.43 (8.25–21.63) ng/mL sepsis vs. 7.23 (5.26–8.82) ng/mL non-sepsis, *p* = 0.004) and the significant level carried on through 24 h after ICU admission (*p* = 0.001) (Figure 2, Table 3).

**Figure 3:**
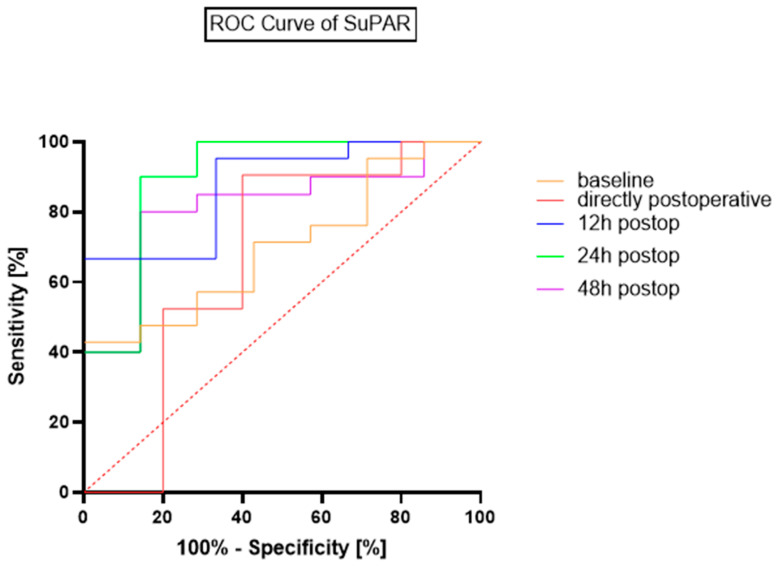
ROC Curves for suPAR’s diagnostic accuracy for sepsis.

ROC-Curve analysis for postoperative suPAR levels revealed high diagnostic accuracy for sepsis during the first 24 h after open repair. The maximum accuracy was reached at 24 h after admission to the ICU. The optimal cut-off point for the suPAR concentration, according to the Youden Index, is 9 ng/mL, which leads to an AUC of 0.91, a sensitivity of 85.7% and a specificity of 90%. At the 24 h postoperative timepoint, the optimal cut-off of 9 ng/mL corresponded to 6 true positives, 1 false negative, 18 true negatives, and 3 false positives. A comparative analysis of the ROCs of the timepoints is presented in Figure 3 and Table 4.

In the Cox proportional hazards model, higher suPAR levels at 12 h after surgery were significantly associated with an increased risk of sepsis (HR = 1.60; *p* = 0.022), indicating approximately a 60% increase in hazard per ng/mL. Longer operation duration was also significantly associated with increased sepsis risk (HR = 1.009; *p* = 0.023), corresponding to approximately 1% increased risk per additional surgery minute. The predictors of the model are displayed in Table 5.

## 4. Discussion

Our study demonstrates that elevated suPAR levels measured as early as 12 h postoperatively are significantly correlated with the subsequent development of sepsis in patients undergoing elective open TAAA repair. In addition, higher suPAR concentrations were associated with prolonged ICU stays, likely reflecting the severity of sepsis and the intensified management required.

Sepsis accounts for up to 30% of all ICU admissions [27] and is associated with a significant increase in mortality and morbidity [28]. Patients undergoing open TAAA repair are particularly vulnerable to sepsis in the early postoperative period due to the pronounced inflammatory response and cytokine activation triggered by the procedure. These processes begin intraoperatively, driven by surgical trauma, the relative ischemia–reperfusion injury during aortic cross-clamping, and the use of cardiopulmonary bypass. Notably, attenuating intraoperative cytokine release has been associated with a tendency of reduced incidence of sepsis in this setting [29].

However, the risk factors for sepsis extend beyond the intraoperative period. In the postoperative phase, patients may experience further inflammatory insults that contribute to a systemic inflammatory response. This disproportionate immune activation leads to excessive secretion of proinflammatory substances, creating an environment of oxidative stress and dysregulated organ perfusion [30]. The related septic state can facilitate the process of systemic disseminated intravascular coagulopathy, which represents a local activation of clotting factors that damage the vascular milieu and can disturb the microcirculation and further organ dysfunction [31,32]. As part of the plasminogen pathways, suPAR is also believed to provide insight into the activation state of coagulation processes [33]. Elevated suPAR levels in patients later diagnosed with sepsis likely indicate a dysregulated immune response, thereby serving as a valuable marker of immune system activation [34].

Given its role in cellular immunological and coagulation activation cascades, processes integral to the development of sepsis, suPAR may be of value in predicting sepsis. Previous studies across various patient populations have demonstrated that early detection of elevated suPAR levels is associated with an increased risk of sepsis, as well as higher mortality and adverse event rates in ICU settings [35,36,37,38,39]. However, there is still a notable literature gap regarding the utility of suPAR in the context of open complex aortic aneurysm surgery. In comparison to other established markers for detecting sepsis, the selected observation interval might allow for earlier stratification of patients at risk of later developing sepsis and aid in the differentiation between the expected immune activation and a dysregulated immune reaction. The latter could lead to sepsis following open TAAA repair [40].

In our patient collective, baseline suPAR levels were not significantly different between the sepsis and non-sepsis groups, with medians of 3.41 and 2.35 ng/mL, respectively. These findings at the preoperative timepoint are consistent with previous literature regarding the average level of serum suPAR in the general population, determined to be 2.36 (2.07–2.81) ng/mL [41]. After surgery, the suPAR levels increased five-fold in the sepsis group to a maximum median concentration of 14.66 (9–22) ng/mL by 24 h postoperatively, subsequently showing a slight decrease at 48 h postoperatively.

The initial increase in concentration following surgery can be explained through the synthesis mechanism of suPAR, which is a glycoprotein released in the bloodstream and other body fluids by a cleavage process from the membrane-bound form of suPAR of various immune cells such as granulocytes and macrophages or endothelial cells [42]. In vivo and in vitro models revealed an increase in concentration of chemokines (e.g., interleukin 8, interleukin 1, tumor necrosis factor a, lipopolysaccharides) that activate nuclear factor kappa b regulated transcriptional pathways and facilitate the synthesis and secretion of suPAR [43]. SuPAR is excreted by the kidneys and might be removed by renal replacement therapy, which could explain the minimal drop in concentration in the sepsis group between 24 h and 48 h postoperatively, from 14.66 to 12 ng/mL [41]. However, because of missing robust data regarding the excretion process of suPAR whilst using renal replacement therapy, we can only speculate on the process observed above.

In the Cox proportional hazards model, higher suPAR levels at 12 h after surgery were significantly associated with an increased risk of sepsis. The same, but to a lesser degree, could be observed for the length of surgery. In this model suPAR levels at 12 h after surgery were the strongest predictor for development of sepsis. The findings underline the possible applicability of suPAR as an early biomarker for sepsis in patients undergoing major aortic surgery.

There are a number of novel postulated approaches meant to aid in the treatment of sepsis. However, the complexity and heterogeneity of the pathophysiology of sepsis impede the development of new therapies. It is of importance to analyze possible biomarkers, such as suPAR which can offer insight into the various pathways that interact in the formation of sepsis, especially in patient groups following major surgery, associated with an increased immunological response [44]. Although promising, the combination of biomarkers in panels fit for regular use in patient care still requires further clinical validation [45,46].

Following the secondary endpoints, one of the main postoperative complications that arise is AKI, with a prevalence of 33% among sepsis patients [47]. Although suPAR has been shown to be able to detect early stages of AKI [48], this trend was not observed in our current study. This could be attributed to the interaction of suPAR with the podocytes of the nephrotic membrane, which is linked to an activation of cell surface integrins, leading to activation of inflammatory cascades and loss of glomerular function [49].

The findings of this retrospective single-center study are subject to several limitations. As open TAAA procedures are becoming internationally rare, the small patient cohort limits the generalizability of our results. The lack of a prospective randomized study design is related to the rarity of the treated disease pattern, causing a relevant risk for selection bias. A further limitation that arises from our study design is the absence of a control group. Thus, we relied on previously published epidemiological data analyzing the concentration of suPAR in the general population. All interventions were performed by a single surgeon, which constrains the generalizability of the results, but also reduces the inter-personal variability of the operating team. Moreover, this approach helps to reduce performance bias associated with the complexity of the procedure administered to the study participants. The small sample size is caused by the rarity of open TAAA repair. Nowadays, only a few centers worldwide perform open repair of TAAA, leading to a severe lack of research in this field of vascular surgery. Although the small number of patients in this study has to be named as a clear limitation regarding the transferability of findings, these could still be considered as reasonable and useful as described. Additionally, chronical impaired renal function may affect SUPAR levels, which has to be mentioned as potential confounder, even if a relevant impact in the assessed cohort could be ruled out.

We hypothesize that routine monitoring of suPAR levels after major vascular surgery, in conjunction with established sepsis diagnostic tools such as blood cultures, may improve the stratification of vulnerable patients and enable earlier initiation of targeted therapy. Despite the robust statistical results, the observational design of our study underscores its hypothesis-generating nature. Consequently, further clinical research is warranted to validate these findings, clarify the clinical significance of suPAR as a biomarker and ultimately enhance patient care for those at risk of sepsis following open TAAA surgery.

## 5. Conclusions

A significant elevation of serum suPAR concentrations in the early postoperative hours in patients receiving open TAAA surgery can be correlated with an increased risk of developing sepsis. Accordingly, routine postoperative suPAR measurements may be valuable in identifying patients at risk of sepsis.

## Figures and Tables

**Table 1:** Demographic characteristics and intraoperative details.

Characteristics	Total N = 28	Sepsis N = 7	No Sepsis N = 21	*p*-Value
Age (years) mean (SD)	52.6 (13.4)	53 (13)	52.5 (13.8)	0.866
Men %	19 (67.9)	4 (57.1)	15 (71.4)	0.646
BMI (KG/m^2^) mean (SD)	25.05 (3.64)	25.47 (3.8)	24.91 (3.68)	0.634
Obesity %	5 (17.9)	2 (28.6)	3 (14.3)	0.574
COPD %	7 (25)	2 (28.8)	5 (23.8)	1
Smoking %	12 (42.9)	4 (57.1)	8 (38.1)	0.418
Diabetes mellitus type 2%	3 (10.7)	2 (28.8)	1 (4.8)	0.145
Hypercholesterolemia %	15 (53.6)	3 (42.9)	12(57.1)	0.67
Atrial fibrillation %	5 (17.9)	2 (28.6)	3 (14.3)	0.574
Heart failure %	4 (14.3)	4 (14.3)	4 (19)	1
Chronic kidney disease %	14 (50)	5 (71.4)	9 (42.9)	0.385
Preexistent hypertension %	22 (78.6)	6 (85.7)	16 (76.2)	1
Marfan %	13 (46.4)	4(57.1)	9 (42.9)	0.67
Aortic OP in the history %	16 (57.1)	5 (71.4)	11 (52.4)	0.662
Stent OP in history %	6 (21.4)	1 (14.3)	5 (23.8)	1
Diameter of aneurysm (cm) mean (SD)	6.46 (1.45)	6.75 (0.52)	6.34 (1.68)	0.102
Type of TAAA				
I	5 (17.9)	0	5 (23.8)	
II	5 (17.9)	1(14.3)	4 (19)	
III	6 (21.4)	0	6 (28.6)	
IV	4 (14.3)	1(14.3)	3 (14.3)	
V	1 (3.5)	0	1 (4.8)	
Operation duration (minutes) mean (SD)	443 (126)	456 (195)	438 (100)	0.015
Time on HLM (minutes) mean (SD)	145 (61)	154 (75)	142 (57)	0.376

**Table 2:** Postoperative outcomes.

Outcomes	Total N = 28	Sepsis N = 7	No Sepsis N = 21	*p*-Value
Mortality %	3 (10.7)	2 (28.6)	1 (4.8)	0.145
Hospital Stay (days) median [IQR]	26.5 [21.25–43.75]	55 [38.5–131.25]	25 [19.5–36]	0.028
ICU stay (days) median [IQR]	15 [7–28.75]	23.5 [15.75–74.75]	9 [6–23.5]	0.01
Time on ventilator (hours) median [IQR]	43 [9–498]	450 [29–1128]	16 [8–283]	0.167
Catecholamine need (days) median [IQR]	3 [1–11]	20 [4.5–27]	2 [1–7]	0.042
Tracheostoma %	8 (28.6)	3 (42.9)	5 (23.8)	0.371
Pneumonia %	15 (53.6)	4 (57.1)	11 (52.4)	1
ARDS %	10 (35.7)	4 (57.1)	6 (28.6)	0.207
Reintervention %	8 (28.6)	4 (57.1)	4 (19)	0.142
Acute kidney injury/Dialysis %	10 (35.7)	4 (57.1)	6 (28.6)	0.207
PostOP delirium%	5 (17.9)	2 (28.6)	3 (14.3)	0.574
Liver failure %	2 (7.1)	1 (14.3)	1 (4.8)	0.444

**Table 3:** Perioperative suPAR levels.

suPAR Concentration ng/mL	Total N = 28	Sepsis N = 7	No Sepsis N = 21	*p*-Value
baseline median [IQR]	2.54 [1.92–4.82]	3.41 [2.32–7.45]	2.35 [1.61–4.22]	0.126
ICU admission median [IQR]	6.3 [4.84–9.08]	11.38 [4.83–15.37]	6.03 [4.82–8.33]	0.278
12 h median [IQR]	8 [5.27–9.19]	14.43 [8.25–21.63]	7.23 [5.26–8.82]	0.004
24 h median [IQR]	7.77 [5.97–9.44]	14.66 [9.01–22.09]	7.05 [4.87–8.05]	0.001
48 h median [IQR]	6.83 [4.97–12.02]	12.02 [8.39–13.32]	6.18 [3.93–7.96]	0.13

**Table 4:** ROC Curve analysis for suPAR and sepsis.

Curve Area Under the ROC					
	Area [95% CI]	*p*-Value	Cut-Off	Sensitivity (%) [95% CI]	Specificity (%) [95% CI]
baseline	0.7 [0.5–0.9]	0.1175	2.136	42.86 [24.47–63.45]	100 [64.57–100]
Directly postop	0.67 [0.32–1]	0.2549	10.8	90.48 [71.09–98.31]	60 [23.07–92.89]
12 h postop	0.87 [0.73–1]	0.0061	9.823	90.48 [71.09–98.31]	66.67 [30–94.08]
24 h postop	0.9 [0.74–1]	0.0019	9	90 [69.9–98.22]	85.71 [48.69–94.92]
48 h postop	0.81 [0.63–0.9]	0.0149	8.2	80 [58.4–91.93]	85.71 [48.69–99.27]

**Table 5:** Cox proportional hazards model.

Predictor	Hazard Ratio	95% CI	*p*-Value
suPAR 12 h postop	1.6	[1.069–2.397]	0.022
Duration of surgery	1.009	[1.001–1.017]	0.023
Delta CRP day 1	0.9	[0.796–1.013]	0.08

## Data Availability

The raw data supporting the conclusions of this article will be made available by the corresponding author, without undue reservation.

## Abbreviations

The following abbreviations are used in this manuscript:

TAAA	Thoracoabdominal Aortic Aneurysm
suPAR	Soluble urokinase Plasminogen Activator Receptors
ELISA	Enzyme-linked immunosorbent assay
ROC	Receiver operating characteristic
AUC	Area Under the Curve
ARDS	Acute respiratory distress syndrome
AKI	Acute kidney injury
MODS	Multiple organ dysfunction syndrome
HLM	Heart–lung machine
ICU	Intensive care unit
CRP	C-reactive protein
